# The microbiome in endometrial cancer: vaginal milieu matters

**DOI:** 10.3389/fmed.2025.1533344

**Published:** 2025-05-09

**Authors:** Igor Govorov, Eduard Komlichenko, Elena Ulrikh, Elena Dikareva, Tatiana Pervunina, Oksana Vazhenina, Amina Dzharbaeva, Olga Kalinina, Ekaterina Zaikova, Zoia Razumova, Miriam Mints, Stanislav Sitkin

**Affiliations:** ^1^Almazov National Medical Research Centre, Saint-Petersburg, Russia; ^2^Division of Obstetrics and Gynecology, Department of Women's and Children's Health, Karolinska Institutet, Stockholm, Sweden; ^3^St Petersburg University, Saint-Petersburg, Russia; ^4^North-Western State Medical University named after I.I. Mechnikov, Saint-Petersburg, Russia; ^5^National Medical Research Centre of Oncology named after N.N. Petrov, Saint-Petersburg, Russia; ^6^School of Medical Science, Faculty of Medicine and Health, Örebro University, Örebro, Sweden; ^7^Institute of Experimental Medicine, Saint-Petersburg, Russia

**Keywords:** endometrial cancer, microbiota, endometrial microbiota, endometrial hyperplasia, carcinogenesis, vaginal microbiota

## Abstract

Endometrial cancer remains one of the most common malignancies in women, and its incidence is particularly increasing in developed countries. Despite the well-known promotive role of excessive exposure to estrogen, many other details of the pathogenesis of endometrial cancer remain unknown. Recent studies have elucidated the emerging role of the resident microbiota in the progression of various diseases, including cancer. Next-generation sequencing demonstrated that the uterine cavity, previously considered sterile, contains a composition-rich microbiota. In this work, we determined the differences in the composition of the intrauterine microbiota between patients with endometrial cancer and its precursor—endometrial hyperplasia.

## Introduction

Endometrial cancer (EC) is the most common gynecological malignancy in developed countries, second only to cervical cancer in the developing part of the world. Unlike many other cancers, the incidence of EC and associated mortality have been increasing in recent decades (1). Most EC cases (around 80%) are histologically endometrioid, present early, and have a more favorable prognosis. These cases are known under the group name Type 1 EC tumors, first described by Bokhman (2). The rest - serous, clear cell, mucinous, and several others - were grouped under the name Type 2 EC tumors. Type 1 tumors usually contain sex steroid receptors and arise from hyperplastic preconditions, such as endometrial hyperplasia (EH). Their development is traditionally related to unopposed exposure to estrogens. The latter may be the consequence of the action of risk factors, including, but are not limited to, obesity, nulliparity, estrogen therapy, etc.

However, in light of new publications and the discoveries of the new genomic atlas, endometrial cancers seem to be more heterogeneous (3). The four distinct subtypes were described: DNA polymerase epsilon ultra-mutated, microsatellite instability hypermutated, copy-number low, and copy-number high. The recent studies suggest that microbiota can also play role in EC tumorogenesis (4, 5). While the vaginal microbiota is well-known to be active and dominated by the *Lactobacilli* genus, the uterine cavity was previously considered sterile (6, 7). The advent of next-generation sequencing (NGS) technologies revealed that the uterine microbiota is more scarce than the vaginal (10^3^-10^5^ times lower), but simultaneously more diverse (8). The origin of the intrauterine microbial community and its role in health and disease is an emerging area of research. For instance, recent studies suggested that the endometrial microbiota can modulate uterine receptivity and therefore influence fertility (9, 10). Furthermore, the composition of the uterine microbiota has been associated with various gynecological conditions, including endometriosis, dysfunctional menstrual bleeding, and cancer (8, 11). The current study was conducted to provide a comparative analysis of the reproductive tract microbiota between patients with EH and EC.

## Materials and methods

### Study group

We included patients who were referred to the Almazov National Medical Research Center from March 2020 to November 2021, due to thickened endometrium on ultrasound and/or abnormal uterine bleeding. The samples were collected and stored prospectively (see below) but were not included in the study before the final histopathological diagnosis. Then the patients were grouped in either of two groups—EH or EC.

Patients were advised against vaginal douching or using vaginal suppositories. None of the patients had previously received menopausal hormone therapy. The exclusion criteria also included tamoxifen use, inflammatory bowel disease, antibiotic use within previous 3 months.

### Sample processing

Samples were collected according to the following method. All procedures were performed in a sterile environment within the operating room by the surgeon. High vaginal swabs (from the posterior fornix) were collected before applying the disinfectants shortly after administering the anesthesia. Routine pre-surgical douching with betadine was then performed. Endometrial samples were collected using disposable tools, Endometrial Tao Brush™ by Coop Medical. It has a protective shield, which helps avoid cross-contamination with the lower parts (vagina, cervical canal) and was previously described as a reliable tool for collecting intrauterine samples (12). The samples were then quickly delivered for storage in liquid nitrogen.

### DNA extraction and 16S rDNA sequencing

Total DNA was extracted using the FlexiGene DNA Kit (Qiagen, Hilden, Germany) following the manufacturer's instructions. DNA quality was assessed using a NanoDrop 1000 spectrophotometer (Thermo Fisher Scientific, Waltham, MA, USA). The DNA was stored at -20°C until use.

Libraries were generated with NEXTflex 16S V4 Amplicon-Seq Kit 2.0 (PerkinElmer, Inc., Waltham, MA, United States), according to the manufacturer's instructions. Sequencing was performed on the MiSeq platform with MiSeq Reagent Kit v2 (250 bp × 2) (Illumina Inc., San Diego, CA, United States).

### Bioinformatic analysis

Files with forward and reverse reads were subjected to primary quality control using the FastQC v0.11.9 (13) and MultiQC v1.14 (14) programs. Filtering of data with low quality value, removal of adapter sequences and primers was performed using the trimmomatic program v0.39 (15) (adapters: 2:30:10 SLIDINGWINDOW: 4:15 HEADCROP (primer length) MINLEN 150). Then a second quality analysis was carried out. The reads were analyzed using the DADA2 package for R v1.26.0 (16). Since the merging of forward and reverse readings led to a large reduction in the number of sequences, only direct readings were used for analysis. Quality control was repeated with the DADA2 package, chimeric reads and low-quality readings were removed. The obtained amplicon sequence variants (ASVs) were clustered using the mmseq2 v 13.45111 (17) with the identity parameter - 99% and 97%, and coverage of 80% to obtain operational taxonomic units (OTUs). The taxonomic analysis of the obtained sequences was carried out in the DADA 2 package based on the SILVA v138.1 database.

Alpha and beta diversity was compared using the phyloseq v1.42.0 package (18). Alpha diversity was evaluated according to the Chao1, Shannon and Simpson criteria. To visually assess beta diversity, a principal coordinate analysis (PCoA) was performed using the Weighted UniFrac algorithm. The LEfSe package microbiomeMarker v1.4.0 (19) was used to detect the bacterial markers that contribute the most to the difference between the two groups.

### Statistics

Statistical analysis was carried out using R packages. Permutational multivariate analysis of variance (PERMANOVA) was performed using the vegan v2 package.6.4 (20), the adonis2() function. Mann-Whitney-Wilcoxon test were used to compare the distribution of the continuous variables between the groups. Chi-square was used to test relationships between categorical variables (together with Yates' correction for continuity when needed).

### Data availability

The 16S rDNA data is available upon request and will be shortly deposited at the NCBI sequence read archive.

### Ethics declaration

The current study was approved by the Institutional Ethical Board No 0306-20 on 15.06.2020. All patients signed informed consent before entering the study.

## Results

### Background characteristics

Both EC and EH groups included 27 patients. EC patients were predominantly older and postmenopausal. There were no differences in severity, parity, or BMI, although the patients in both groups tended to be overweight (Table 1). Within the EC group patients mostly had localized type I cancer, except one patient, who had serous carcinoma.

**Table 1:** Background patients' characteristics.

		**EC group (*n* = 27)**	**EH group (*n* = 27)**	***P*-value**
Age, years Median ± IQR		65.00 [59.00, 69.00]	55.00 [45.50, 60.00]	0.002*
BMI Median ± IQR		32.80 [27.45, 39.50]	29.00 [25.25, 32.70]	0.063
Menopause, *n* (%)		24 (88.9)	16 (59.3)	0.030*
Number of pregnancies, Mean (SD)		3.00 (2.42)	3.00 (2.48)	1.00
Number of childbirth, Mean (SD)		1.48 (1.37)	1.33 (1.00)	0.652
Stage, *n* (%)	IA	14 (51.9)	-	-
	IB	6 (22.2)	-	-
	II	3 (11.1)	-	-
	IIIA	1 (3.7)	-	-
	IIIC1	1 (3.7)	-	-
	IIIC2	1 (3.7)	-	-
	IVB	1 (3.7)	-	-
	Grade, n (%)	G1	11 (40.7)	-	-
		G2	6 (22.2)	-	-
		G3	10 (37.0)	-	-

BMI, body mass index; EC, endometrial cancer; EH, endometrial hyperplasia; IQR, interquartile range; *statistically significant.

### Uterine microbiota composition

**Figure 1:**
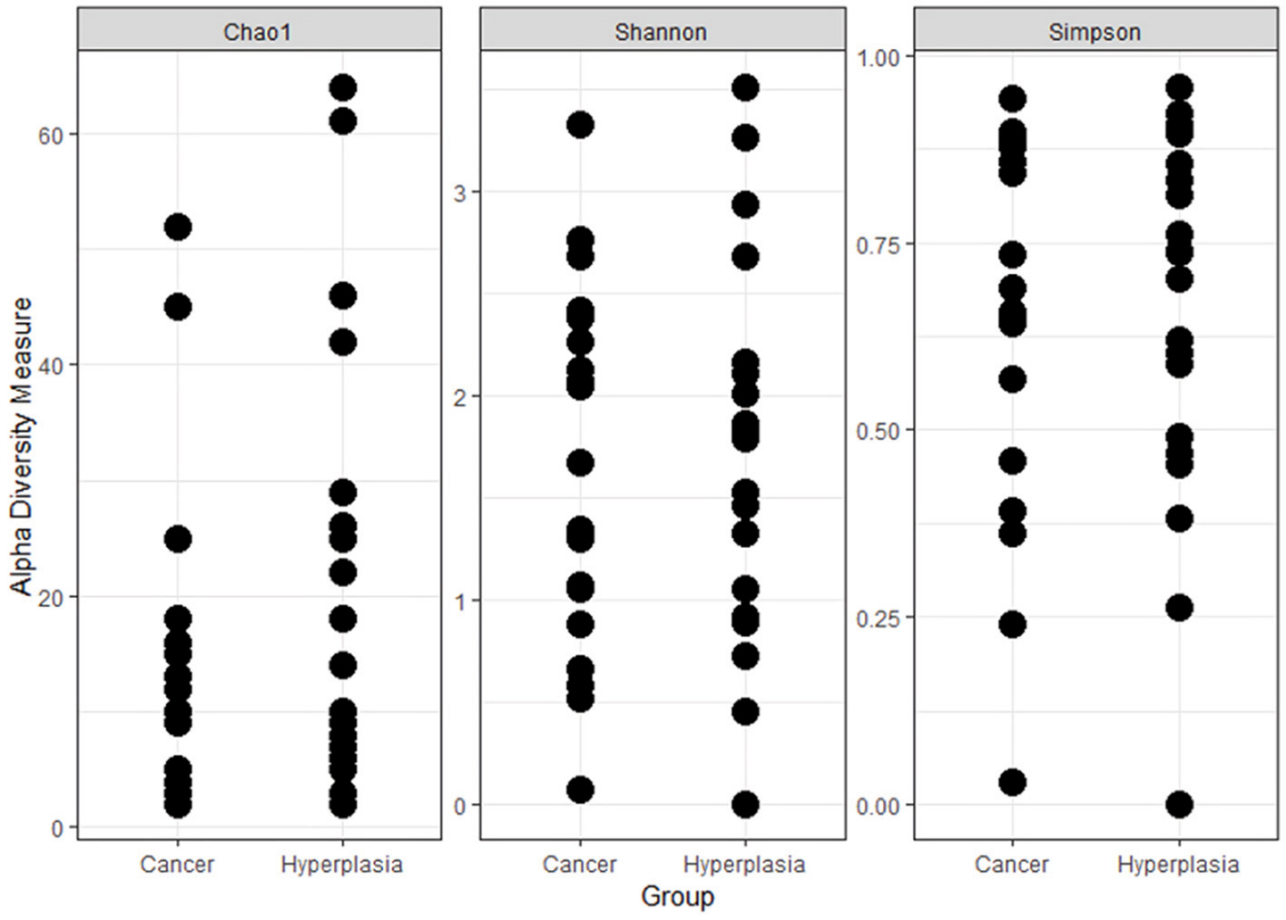
Alpha-diversity of the uterine samples according to different metrics.

In total 49 uterine samples were obtained (5 were discarded due to unsatisfactory quality). After potentially human sequences were subtracted, the number of reads equaled zero in two samples. Therefore, 47 samples were included in the final analysis, with 24 and 23 in the EC and EH groups, respectively. Figure 1 shows the alpha-diversity of the uterine samples, according to Chao1, Shannon and Simpson respectively.

**Figure 2:**
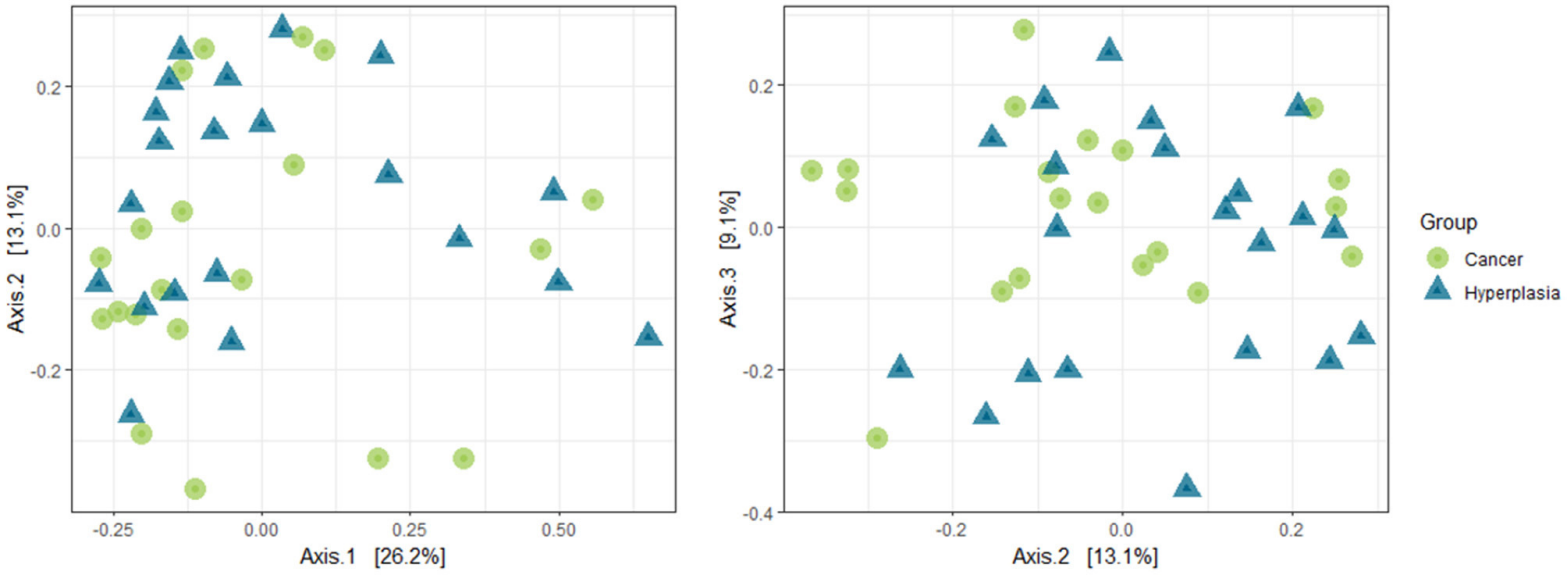
Beta-diversity of the uterine samples in 1:2 and 2:3 projections.

The intergroup diversity between two groups was analyzed using PCoA and weighted Unifrac algorithms. The results are presented in Figure 2. Additionally, PERMANOVA was used to analyze the data. As a result, a *p*-value of 0.006 was obtained, indicating a statistical difference between the two groups.

**Figure 3:**
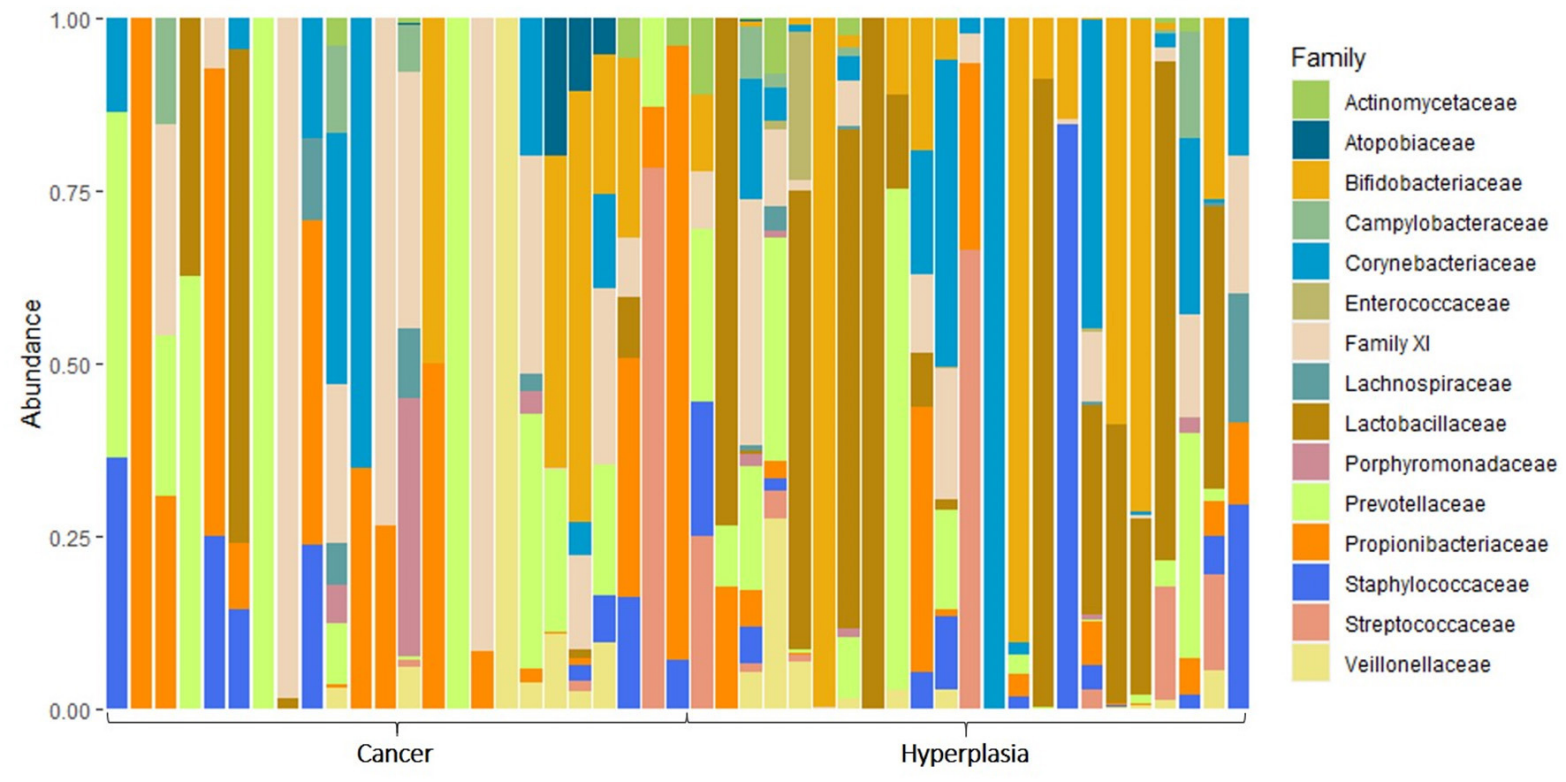
The taxonomic composition of uterine microbiota samples at the level of bacterial families.

When analyzing the taxonomic diversity of the uterine microbiome, 15 different families of bacteria were identified (Figure 3). The most prevalent families included *Porphyromonadaceae, Prevotellaceae, Actinomycetaceae*, and *Enterococcaceae*.

**Figure 4:**
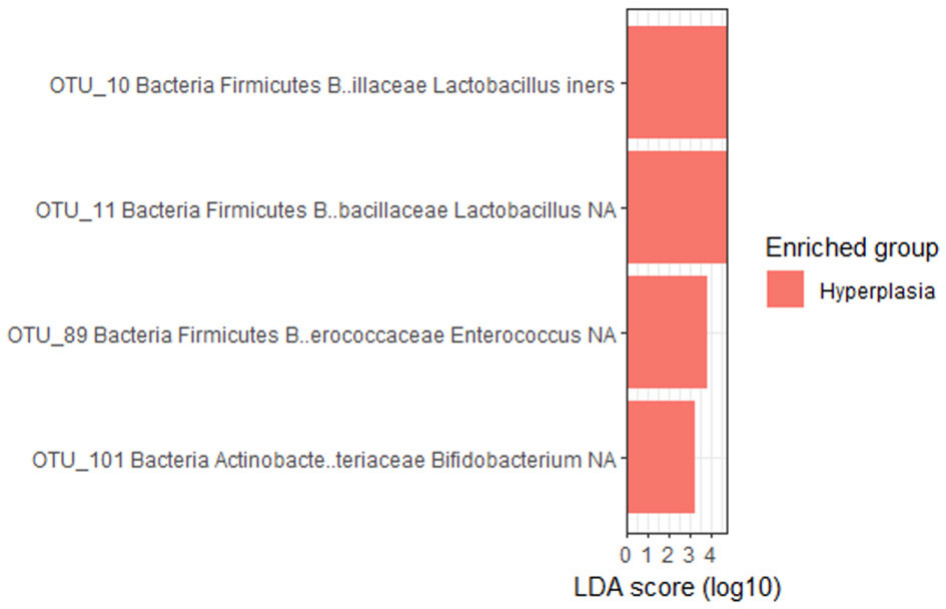
Bacterial agents, that were enriched in EH group, according to LEfSe analysis.

The LEfSe method revealed that four intrauterine bacterial agents were enriched in EH group and by that contributed the most to the differences between the groups (Figure 4). These were *Lactobacillus iners*, and unknown species of the genera *Lactobacillus, Enterococcus* and *Bifidobacterium* genera.

### Vaginal microbiota composition

**Figure 5:**
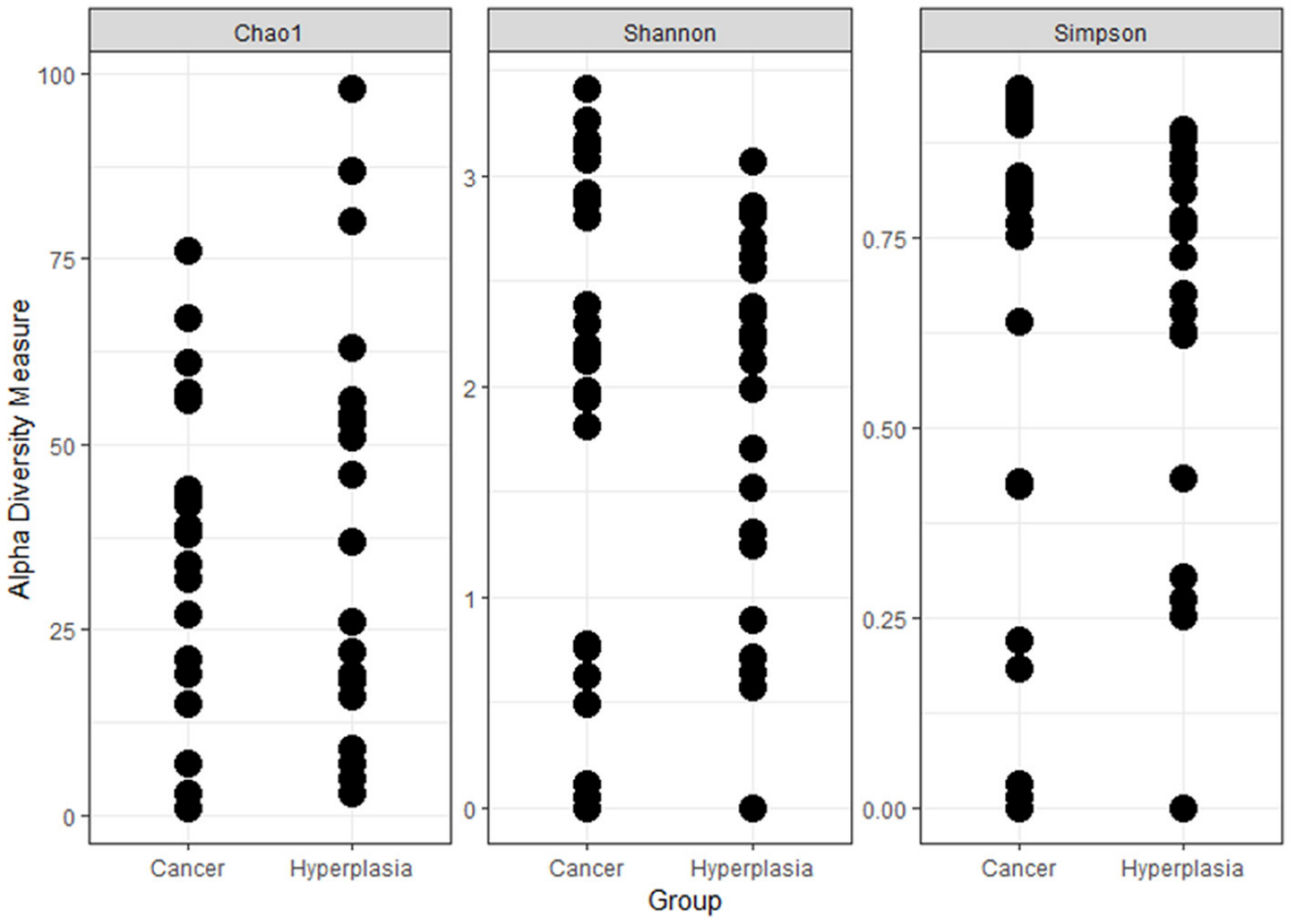
Alpha-diversity of the vaginal samples according to different metrics.

In total 44 vaginal samples were obtained (excluding those with preanalytical flaws), of which 23 belonged to the EC group and 21 belonged to the EH group. Figure 5 shows the alpha diversity of the vaginal samples, according to Chao1, Shannon, and Simpson, respectively.

**Figure 6:**
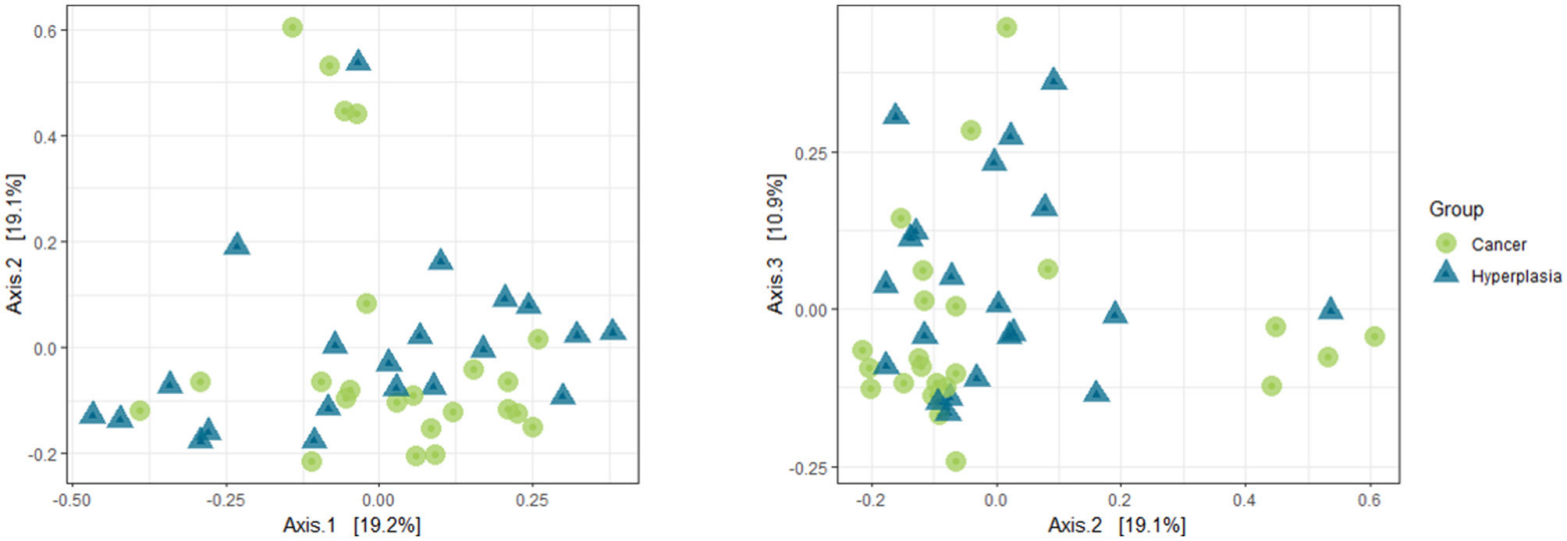
Beta-diversity of the vaginal samples in 1:2 and 2:3 projections.

The intergroup diversity for the vaginal samples is presented in Figure 6. The PERMANOVA analysis revealed a *p*-value of 0.01. This indicates that the two sets of samples statistically differ from each other in terms of beta diversity.

**Figure 7:**
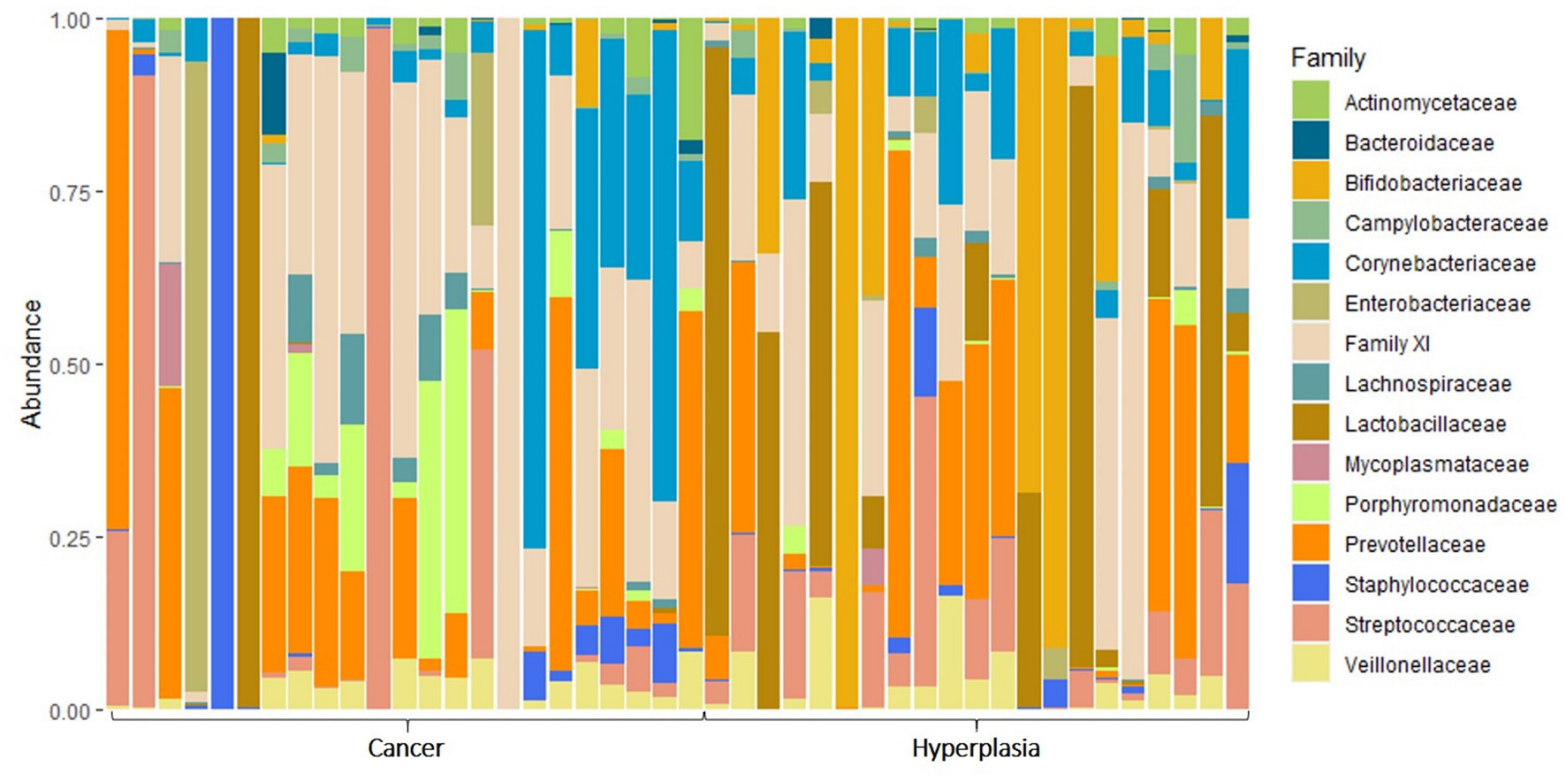
The taxonomic composition of vaginal microbiota samples at the level of bacterial families.

Taxonomic analysis revealed the presence of 26 different classes of bacteria. Figure 7 illustrates the top-15 families. The most prevalent families were *Prevotellaceae (Bacteroidota), Bifidobacteriaceae (Actinomycetota), Porphyromonadaceae (Bacteroidota)*, and *Corynebacteriaceae (Actinomycetota)*.

**Figure 8:**
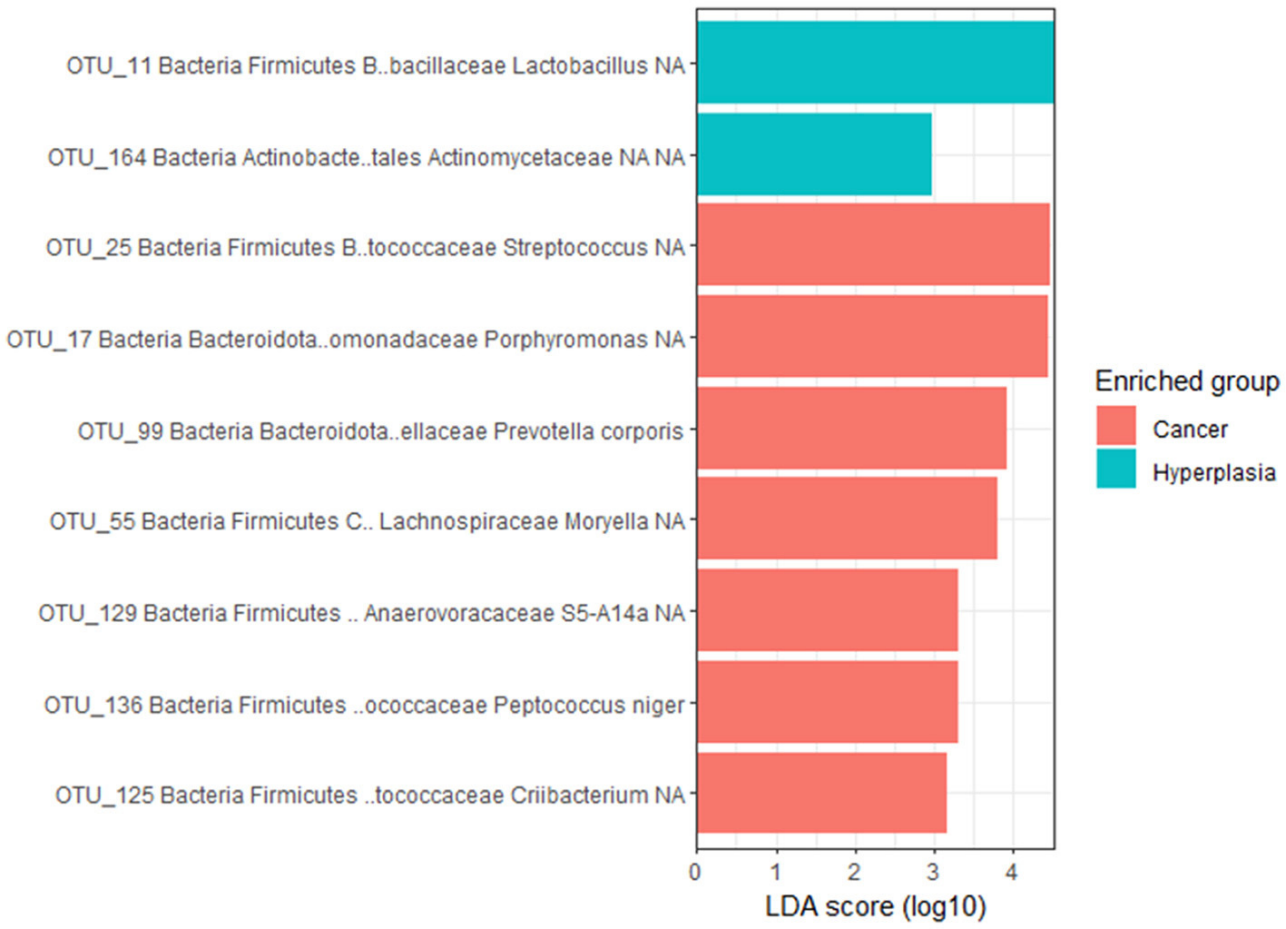
Comparison of bacterial agents, that were enriched in vagina in both groups.

The LefSe method identified nine biomarkers, two of which were enriched in EH group—*Lactobacillus (Bacillota)* and *Actinomycetaceae (Actinomycetota)* and seven - in EC group (Figure 8). The latter included *Porphyromonas spp, Prevotella corporis* (both *Bacteroidota*); *Streptococcus spp, Moryella spp, Peptococcus niger, Criibacterium spp* and *Clostridiales* bacterium S5 A14a (all belong to *Bacillota*).

**Figure 9:**
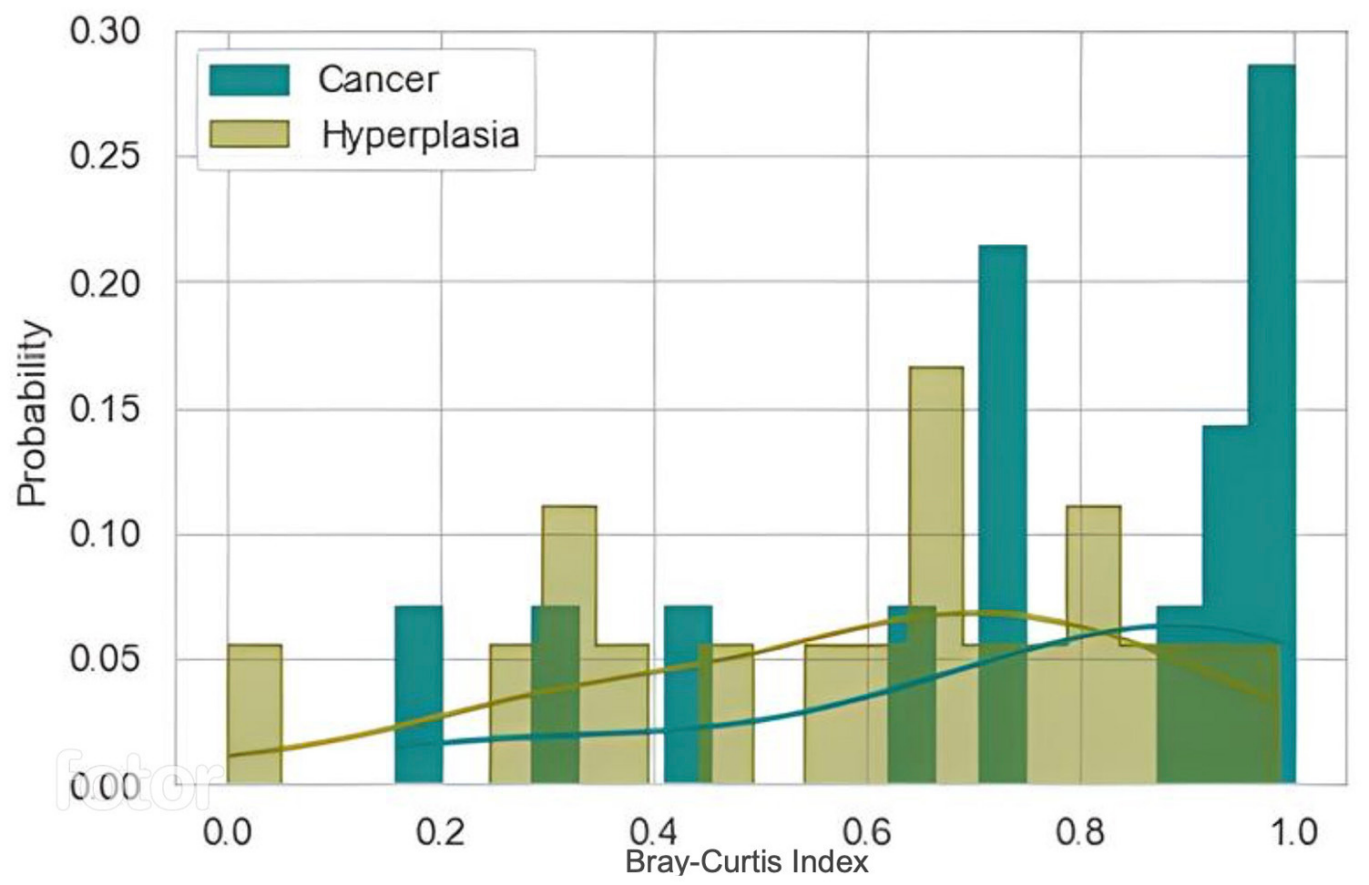
Pairwise samples comparison using Bray-Curtis distance metric.

After excluding samples with low number of reads, 32 samples were analyzed pairwise (14 in Cancer group, 18 in Hyperplasia group). Paired samples with hyperplasia were more similar to each other than paired samples in the cancer group (Figure 9). However, *p*-value was 0.1315, therefore not allowing us to draw a definitive conclusion.

## Discussion

In the current study we investigated differences in microbiota composition both in the vagina and the uterus between EH and EC patients. The results indicate the presence of a scarce but diverse microbial community within the uterus, as shown by several intragroup diversity parameters, the Chao1, Shannon, and Simpson index. Furthermore, both the vaginal and endometrial microbial composition *per se* differs between the groups with possible explanations and implications to be described below.

The existence of bacteria in the uterus has long been a controversial topic, with early research relying on culture-based methods suggesting the uterus was sterile. This may be due to the imperfections of the method, as well as the fact that some bacteria are known to grow poorly on artificial media. However, recent culture-independent studies using next-generation sequencing have revealed the presence of diverse microbial communities in the uterus, referred to as the uterine microbiota (21). In healthy women, the uterine microbiota appears to be low in biomass, but highly diverse, containing bacteria belonging mainly to the *Lactobacillus (Bacillota), Prevotella (Bacteroidota), Gardnerella*, and *Atopobium* (both belong to *Actinomycetota*) (22). The specific composition of the microbiota varies according to factors such as the menstrual cycle, parity and menopausal status (23). In postmenopausal women, the uterine mileue was enriched in Flavobacterium (Bacteroidota) compared to the cervicovaginal and anorectal microbiota (24). In contrast, in nulliparous women with abnormal uterine bleeding *Prevotella, Fusobacterium* and *Jonquetella* were the most abundant taxa (25). Infertility, endometriosis, and oncogynecological diseases are among the most studied areas in the context of microbial influence (26–28).

The uterus is an immunologically priveleged organ: it can accommodate tissue invasion by immunologically semiforeign placental cells, yet maintain mucosal immune defenses against ascending foreign organisms, and provide a system to efficiently clear the endometrial detritus that results from menstruation. The relations between the human body and the resident microbiota are an emerging scientific topic, which has not yet been described. Sometimes, the microbe-host interactions are simplified by saying that these two entities apply oppositely directed forces: The microbes strive to thwart host defense and multiply infinitely, while the host puts all efforts into eradicating the microorganisms. Although this might be acceptable or at least pragmatic for using this model for acute infections, the pattern of how the host interacts with the resident microbiota is not linear and is best described by the Nash dynamic equilibrium (29). In such a model, a host sequesters a microbe community within defined locus (should that be the enteric lumen or endometrial cavity) and does not favor their spread beyond these borders.

In our study members of both *Porphyromonadaceae* and *Prevotellaceae* families were among the most prevalent bacteria in the vagina and the uterus. This is in accordance with the previous studies. For example, the study by Caselli et al. showed that both *Atopobium vaginae* and *Porphyromonas somerae* induced the expression of pro-inflammatory cytokines in endometrial cells, which could have implications for the development of endometrial cancer (30). Based on sequencing of the 16S rDNA V3-V5 region from the reproductive tract, Walther-António et al. suggest that the detection of *Atopobium vaginae* and *Porphyromonas sp*. in the gynecologic tract combined with a vaginal pH greater than 5, had a sensitivity of 100% and specificity of 60% to detect EC (31). In a recent study by the same group (32) *Porphyromonas* was described as a robust biomarker of EC. *Porphyromonas uenonis* was more common in vaginal specimens from patients with both high-grade and low-grade endometrial cancers than from those with benign conditions (33). Wang et al. studied the microbiota in EC and adjacent tissue samples and found that several genera were enriched in malignant loci—*Porphyromonas, Prevotella, Atopobium, Anaerococcus, Dialister*, and *Peptoniphilus* (5). Li et al. provided evidence that tumor burden is associated with the presence of a specific endometrial microbiota, which is distinguished by the enrichment of *Pelomonas* and *Prevotella* in patients with EC (34). Transcriptomic data identified the upregulation of eight genes involved in pathways associated with fibrin degradation, including those with proteolysis and transcription factor activities. Hence, the authors concluded that *Prevotella* may promote fibrin degradation by inducing the expression of specific genes. It should be also noted, that *Porphyromonas* was previously described as carcinogenic within the digestive tract, due to its ability of inducing immune responses in the host (35).

In the current study, potential biomarkers for endometrial cancer have only been identified in the vaginal microbiota. Typically absent from the normal vaginal microbiota, including *Porphyromonas, Prevotella corporis, Streptococcus, Moryella, Peptococcus niger, Criibacterium* and *Clostridiales bacterium S5 A14a*. Notably, vaginal *Porphyromonas* and *Prevotella*, along with *Bacteroides, Mycoplasma, Bacillus, Dialister*, and *Anaerococcus*, have recently been identified as indicators of cervical cancer (36). *Prevotella corporis*, an established human pathogen, along with *Prevotella bivia* and *Prevotella disiens*, originates from the urogenital tract (37), but can also be an odontogenic pathogen (38). *Prevotella corporis*, an anaerobic bacterium rarely mentioned in the literature, was previously found to be associated with bacterial vaginosis, including in pregnant women (39), and has never been reported to be associated with EC until the current study. However, other *Prevotella* species and the *Prevotella* genus in general have previously been found to be associated with various gynecological cancers. *Prevotella bivia* was one of two species, along with *Fusobacterium ulcerans*, significantly more abundant in high grade compared to low grade endometrial carcinoma (33), and even earlier was found associated with cervical cancer (40). As previously shown, the abundance of vaginal *Prevotella* and *Streptococcus* was increased in the group of gynecological cancers (41). Although *Prevotella* spp. generally cause minimal proinflammatory epithelial activation, they may induce changes to the cellular physiology and integrity of the endometrial epithelium in a species-specific fashion. *Prevotella* spp. can form biofilms, alter the epithelial barrier, and impact the colonization of secondary colonizers (42).

*Moryella*, currently represented by a single species *Moryella indoligenes*, is a minor component of the vaginal microbiome (43), closely correlated with bacterial vaginosis (44). This bacterium, along with *Prevotella*, was previously described as being abundant in the vaginal samples from the females with EC (45). The association with gastrointestinal cancer was also reported (46).

*Peptococcus niger*, is an established human pathogen, previously found in a healthy vaginal ecosystem (47). This species has not previously been reported to be associated with gynecological cancers, however, it was previously identified in oral swabs in patients with potentially malignant disorders (48).

*Criibacterium* a genus with a single specie *Criibacterium bergeronii* isolated from human clinical samples, was shown to be enriched in the EC tissues, as mentioned in the work by Wang et al. (5). *Criibacterium bergeronii*, besides *Streptococcus pyogenes*, was the only other bacterial species enriched in idiopathic cutaneous ulcers in children after community-based mass treatment with azithromycin (49).

*Clostridiales bacterium S5 A14a* and *Anaerovoracaceae* family to which this bacterium probably belongs were found to be abundant in vaginal swabs in patients who had previous malignancies (50). In addition, *Clostridiales bacterium S5-A14a* was one of the taxa differentiating healthy control and post-treatment cervical swab samples in premenopausal women with squamous cell carcinoma of the cervix (51).

Intriguingly, the genes of *Porphyromonas, Prevotella, Streptococcus*, and *Peptococcus niger* can be classified as part of the estrobolome. The first three taxa can encode β-galactosidases, and *Peptococcus niger* can encode sulfatases and convert estrone sulfate to estrone, influencing active estrogen levels - the major risk-factor for EC (52, 53). In a recent study, for example, changes in gut *Porphyromonas* and *Prevotella corporis* correlated with postmenopausal hot flashes, which may also be related to their effect on estrogen levels (54).

Limitations of the study include the limited number of samples and differences in age and menopausal status between the groups. On the one hand, it reflects the natural distribution observed clinically, as patients with endometrial cancer tend to be older than those with hyperplasia (55, 56). In particular, Walsh et al. (32) also reported that postmenopausal status and age were significant modifiers of the microbiome composition. At the same time, we believe that, since the results of our research are consistent with previous studies, especially regarding *Porphyromonas* and *Prevotella*, menopause should be considered as only one of several factors that influence the outcome. The hypoestrogenism typically observed in postmenopause might potentially modulate the microbiota through interactions betwen estrogen and intraepithelial glycogen. The latter serve as a substrate for *Lactobacilli*, which act as antagonists to other bacteria through producing hydrogen peroxide and antimicrobial compounds. This interaction becomes even more complex when considering that multiple bacterial species can affect estrogen reabsorption and enterohepatic circulation by their β-glucuronidase and β-glucosidase enzymes that deconjugate estrogens (57, 58). Therefore, it can be hypothesized that increased activity of the deconjugating bacteria could lead to increased estrogen levels, a known risk factor for EC.

In conclusion, both vaginal and endometrial microbiota are different in terms of composition between patients with EH and EC. Several taxa were enriched in vaginal samples in patients with EC: *Prevotella corporis, Porphyromonas, Streptococcus, Moryella, Criibacterium, Peptococcus niger and Clostridiales bacterium S5-A14a*. We believe that these differentiating taxa found may be a hallmark of EC-associated vaginal dysbiosis. Our results therefore support previous findings suggesting differences in the composition of the genital tract microbiome, which could potentially be used for the diagnosis of EC, including the differential diagnosis of EH.

## Data Availability

The 16S rRNA data were deposited at the NCBI sequence read archive under the BioProject PRJNA1255609.
